# Cost-Effectiveness Analysis of Endoscopic Ultrasound versus Magnetic Resonance Cholangiopancreatography in Patients with Suspected Common Bile Duct Stones

**DOI:** 10.1371/journal.pone.0121699

**Published:** 2015-03-23

**Authors:** Stephen Morris, Kurinchi S. Gurusamy, Jessica Sheringham, Brian R. Davidson

**Affiliations:** 1 Department of Applied Health Research, University College London, Gower Street, London, United Kingdom; 2 Department of Surgery, University College London Medical School, 9th Floor, Royal Free Hospital, Rowland Hill Street, London, United Kingdom; University of Calgary, CANADA

## Abstract

**Background:**

Patients with suspected common bile duct (CBD) stones are often diagnosed using endoscopic retrograde cholangiopancreatography (ERCP), an invasive procedure with risk of significant complications. Using endoscopic ultrasound (EUS) or Magnetic Resonance CholangioPancreatography (MRCP) first to detect CBD stones can reduce the risk of unnecessary procedures, cut complications and may save costs.

**Aim:**

This study sought to compare the cost-effectiveness of initial EUS or MRCP in patients with suspected CBD stones.

**Methods:**

This study is a model based cost-utility analysis estimating mean costs and quality-adjusted life years (QALYs) per patient from the perspective of the UK National Health Service (NHS) over a 1 year time horizon. A decision tree model was constructed and populated with probabilities, outcomes and cost data from published sources, including one-way and probabilistic sensitivity analyses.

**Results:**

Using MRCP to select patients for ERCP was less costly than using EUS to select patients or proceeding directly to ERCP ($1299 versus $1753 and $1781, respectively), with similar QALYs accruing to each option (0.998, 0.998 and 0.997 for EUS, MRCP and direct ERCP, respectively). Initial MRCP was the most cost-effective option with the highest monetary net benefit, and this result was not sensitive to model parameters. MRCP had a 61% probability of being cost-effective at $29,000, the maximum willingness to pay for a QALY commonly used in the UK.

**Conclusion:**

From the perspective of the UK NHS, MRCP was the most cost-effective test in the diagnosis of CBD stones.

## Introduction

Patients with suspected common bile duct (CBD) stones are usually diagnosed using endoscopic retrograde cholangiopancreatography (ERCP), which is considered to be the reference standard.[1] Using ERCP for CBD stone diagnosis means that stones can be removed during the same procedure following endoscopic sphincterotomy (ES). ERCP is an invasive procedure, which has a risk of major complications such as pancreatitis and cholangitis associated with morbidity and mortality.[2] The prevalence of CBD stones among patients at risk or suspected of having this problem is 14–68%,[3–20] indicating that 32–86% of these patients are unnecessarily undergoing a procedure with a risk of major complications.

Endoscopic ultrasound (EUS) and magnetic resonance cholangiopancreatography (MRCP) can be used to identify patients likely to have CBD stones. Patients can then proceed to invasive testing and treatment by ERCP. Unnecessary invasive testing can be avoided in patients who on the basis of EUS or MRCP are unlikely to have CBD stones which may reduce the costs and negative impact on patient health of invasive testing. However both tests incur costs and neither is 100% accurate.

A recent Cochrane Review found that use of EUS or MRCP prior to ERCP can potentially reduce the rate of unnecessary invasive testing by 30–70%.[21] A review of the NHS Economic Evaluations Database using the search term “bile duct” [31 August 2013] identified 24 studies relating to cost-effectiveness.[22] Howard et al found MRCP was less costly and more effective than ERCP in patients with abdominal pain and/or abnormal liver function tests at a prior probability of CBD stones of <60%.[23] Kaltenthaler et al found that MRCP was less costly and more effective than ERCP in patients with biliary tree obstruction.[24–25] Arguedas et al found that in patients with acute biliary pancreatitis at probabilities of CBD stones of ≤45% EUS was most cost-effective, and at probabilities >45% direct ERCP was most cost-effective.[26] None of these evaluated EUS versus MRCP versus invasive testing with ERCP in patients with suspected CBD stones. Hence the relative costs and benefits of EUS, MRCP and direct invasive testing are unclear.

This study aimed to investigate the cost-effectiveness of EUS versus MRCP in patients with suspected CBD stones.

## Methods

This is a model-based cost-utility analysis to estimate the mean cost and outcome per patient associated with EUS versus MRCP prior to ERCP compared with proceeding directly to ERCP in patients with suspected CBD stones based on symptoms such as obstructive jaundice, pancreatitis, abnormal ultrasound or liver function tests. The outcome measure is quality-adjusted life years (QALYs), which combine length and quality of life.[27] QALYs are the recommended outcome for use in UK economic evaluations.

The analysis is undertaken from the perspective of the UK National Health Service (NHS). Costs are calculated in 2011/12 UK£ and presented in US$ using an exchange rate of US$1 = £0.69.[28] Since EUS or MRCP are unlikely to affect long term disease outcomes, a time horizon of 12 months for costs and outcomes was considered to be appropriate and discounting of costs and benefits unnecessary.

### Model structure

**Fig 1:**
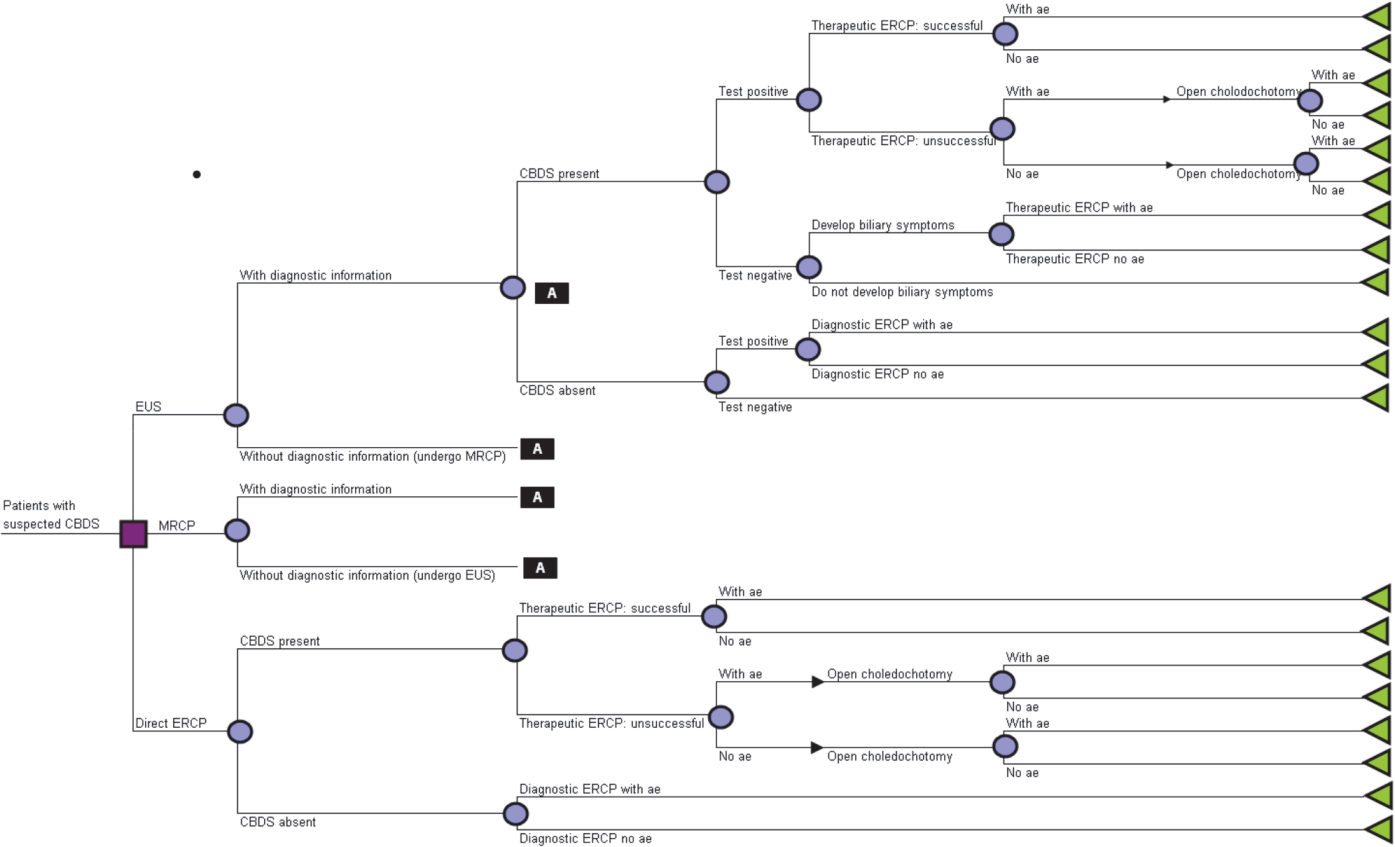
Decision tree model structure. Description of the decision tree options compared. The nodes are points where more than one event is possible. Decision nodes (represented by squares) show the different options that might be chosen by decision-makers. Chance nodes (circles) show uncertain events, each of which is associated with a probability that it will occur. The branches are mutually exclusive events following each node. Terminal nodes (triangles) are the decision tree endpoints, beyond which no further pathways are available. CBDS = common bile duct stones. EUS = Endoscopic ultrasound. MRCP = magnetic resonance cholangiopancreatography. ERCP = endoscopic retrograde cholangiopancreatography. c = complications.

The analysis uses a decision tree to describe the options compared (Fig. 1). This is a commonly used approach in cost-effectiveness studies of healthcare programmes.[27] The decision tree nodes are points where more than one event is possible. The branches are mutually exclusive events following each node. Decision nodes (represented by squares) show the different options that might, in theory, be chosen by decision-makers (e.g., to choose EUS, MRCP or direct ERCP). We are primarily interested in the comparison between EUS and MRCP, using direct ERCP as a benchmark. Chance nodes (circles) show uncertain events, each of which is associated with a probability that it will occur (e.g., whether EUS and MRCP will give positive test results or not). Terminal nodes (triangles) are the decision tree endpoints, beyond which no further pathways are available. Each terminal node has costs and QALYs associated with it, summarising the sequence of decisions and events on a unique path from the initial decision node to that terminal node. These costs and QALYs are expected values, based on the probability of each event on the pathway occurring up to that point and the costs and QALYs associated with each event.

Patients suitable for EUS or MRCP with no contraindications for endoscopic clearance of CBD stones enter the model at risk of or suspected to have CBD stones. This may be with or without prior diagnosis of cholelithiasis, with or without symptoms of CBD stones, with or without prior treatment for CBD stones, and before or after cholecystectomy. The model assumes that patients undergo EUS or MRCP as a diagnostic test followed by ERCP if the test indicates the presence of CBD stones. If CBD stones are confirmed by ERCP then the stones are removed during the same procedure following endoscopic sphincterotomy (ES) (called therapeutic ERCP). If stones are absent then ERCP was diagnostic only (diagnostic ERCP). If therapeutic ERCP is unsuccessful in clearing stones endoscopically, patients undergo a second attempt at endoscopic clearance with therapeutic ERCP, failing which they undergo open choledochotomy to remove the stones. If CBD stones are present but not detected by EUS or MRCP (false negatives) then patients may or may not subsequently develop biliary symptoms; if they do, they will undergo ERCP and ES. If EUS or MRCP do not provide sufficient diagnostic information (e.g. due to patient intolerance of the procedure or technical factors such as presence of a pacemaker), it is assumed patients undergo the other test (i.e. EUS in patients who cannot undergo MRCP, MRCP in patients who cannot undergo EUS).

For patients proceeding directly to ERCP it is assumed they undergo diagnostic ERCP, and if CBD stones are present they are removed by therapeutic ERCP or by open choledochotomy if therapeutic ERCP is unsuccessful after the second attempt. If stones are absent patients undergo diagnostic ERCP only. All procedures other than MRCP may have complications.

### Probabilities

The probabilities associated with mutually exclusive events at each chance node were obtained from published sources (S1 Table).[2,21,24,29] The probability that CBD stones were present (the pre-test probability of CBD stones) was estimated as 0.418, which was the median pre-test prevalence of CBD stones across the 18 studies in the Cochrane Review (range: 0.144–0.679).[21] This probability was varied in sensitivity analyses, to reflect the extent of real-life variation.

The Cochrane Review also calculated the sensitivity and specificity of EUS (0.95 (95%CI 0.91–0.97) and (0.97 (95%CI 0.94–0.99)) and MRCP (0.93 (95%CI 0.87–0.96) and 0.96 (95%CI 0.900.98)).[21] The model assumes the sensitivity and specificity of direct ERCP were both 1. The probability of successful endoscopic clearance of stones by therapeutic ERCP was assumed to be 0.989 based on a systematic review,[2] and the probability of complications with therapeutic ERCP (0.187) and open choledochotomy following an unsuccessful first therapeutic ERCP (0.205) were both taken from another Cochrane Review.[29] A UK audit of ERCP found success rates of 60–70%; the impact of this is investigated in sensitivity analyses.[30] The probability of complications associated with diagnostic ERCP (0.055) was taken from a systematic review and meta-analysis incorporating an economic evaluation.[24] The model also accounts for the small probability (0.0003) that EUS may result in complications.[31] There are no published figures for the probability that diagnostic information is not available from EUS or MRCP, so we assume a value of 0.01, varying this from 0 to 0.02 in sensitivity analyses. Limited evidence suggests that asymptomatic bile duct stones may not cause biliary problems.[32] We assume the probability such problems will develop in patients where CBD stones are present but EUS or MRCP give negative results (false negative test results) is 0.01, varying this in sensitivity analyses.

### Outcomes

QALYs combine length of life and quality of life (the latter measured by utility scores). Utility scores of 1 and 0 represent full health and death, respectively; negative values represent states worse than death. A search of the CEA Registry at the Tufts Medical Center was undertaken using the search term “bile duct stones” [31 August 2013] to identify studies reporting relevant utility scores.[33] After reviewing the reference lists of identified studies and removing duplicates, four studies containing relevant utility data were identified.[23,25,34–35] Utility scores for the health states required by the model were taken from one study,[23] selected because utility scores for all the health states in the model were included in this study enabling better comparability between values, and because the study reported parameter values that could be used in sensitivity analyses (S1 Table). No published studies were found that reported on the duration of each event in the decision tree so all assumptions were based on clinical opinion and varied in sensitivity analysis (S1 Table). The duration of each pathway in the decision tree was calculated by summing the duration of each event in that pathway. Patients were assumed to be in full health (i.e. a utility of 1) during the period from the end of the pathway up to 12 months (the time horizon of the model).

### Costs

The costs of EUS with and without complications were assumed to be $1336 and $803 respectively, and the cost of MRCP was assumed to be $356 (S1 Table).[36] The cost of EUS is the average value of the national mean cost of diagnostic endoscopic upper gastrointestinal tract procedures performed on an elective inpatient and day case basis in the UK, weighted by the proportion of patients in each group. The cost of MRCP ($356) is the average value of the national mean cost of magnetic resonance imaging scans for two to three areas, with contrast, performed either on an outpatient basis or as direct access, weighted by the proportion of patients in each group. Diagnostic ERCP was assumed to cost $5601 with complications and $1149 without complications; therapeutic ERCP was assumed to cost $5601 with complications and $1412 without complications.[36] Open choledochotomy was assumed to cost $7794 and $6503 with and without complications.[36] The model accounts for the probability (0.023) that therapeutic ERCP may require a second procedure to successfully remove the CBD stones.[2]

### Measuring cost-effectiveness

Cost-effectiveness was measured using monetary net benefits (MNBs). For each treatment the MNB was calculated as the mean QALYs per patient accruing to that treatment multiplied by decision-makers’ maximum willingness to pay for a QALY (also referred to as the cost-effectiveness threshold) minus the mean cost per patient for the treatment. In the UK the lower and upper limit of the maximum willingness to pay for a QALY are £20,000 ($29,000) and £30,000 ($43,000) respectively.[37] This approach converts the outcomes from each treatment into monetary terms and then subtracts the costs of each treatment from the monetised benefits, calculating the net benefit of each treatment in monetary terms. MNBs were calculated using the base case parameter values (S1 Table); these are referred to as deterministic results since they do not depend on chance. The option with the highest MNB represents good value for money and is preferred on cost-effectiveness grounds.

### Sensitivity analyses

One-way sensitivity analysis was undertaken, varying the probabilities, outcomes and costs one at a time within the ranges in S1. The aim was to identify the values where each option (EUS, MRCP, direct ERCP) had the highest MNB. We undertook a further univariate sensitivity analysis in which the probability of developing biliary symptoms where CBD stones are present but EUS or MRCP give (false) negative results was allowed to vary separately for EUS and MRCP between 0 and 1.

We also undertook a probabilistic sensitivity analysis (PSA).[38] Distributions were assigned to parameters (S1 Table) to reflect the uncertainty with each parameter value. A random value from the corresponding distribution for each parameter was selected. This generated an estimate of the mean cost and QALYs and the MNB associated with each treatment. This was repeated 5000 times and the results for each simulation were noted. The mean value for each model parameter and the mean MNB for each treatment was calculated from the 5000 simulations; these are referred to as probabilistic results since they depend on chance. Using the MNBs for each of the 5000 simulations, the proportion of times each treatment had the highest MNB was calculated for a range of values for the maximum willingness to pay for a QALY. These were summarised graphically using cost-effectiveness acceptability curves.[27]

In the PSA, beta distributions were used to model uncertainty in probabilities and utility scores, and gamma distributions to model uncertainty in costs (S1 Table).[38] Uniform distributions were used where the base case value was based on assumption, and for the probability of complications with EUS, which was reported in a single secondary data source containing a point estimate but no measure of uncertainty.[31] Where standard errors were required for the PSA but not reported, it was assumed these were equal to the mean.[38] For the probability that CBD stones were present (the pre-test probability of CBD stones), the parameter values for the beta distribution were based on the total numbers of patients with and without CBD stones pooled across all studies in the Cochrane Review. The parameter values for the sensitivity and specificity of EUS and MRCP were calculated from the 95% confidence intervals reported in the Cochrane Review.[21] The 95% confidence intervals around the base case values were derived using standard deviations calculated from the 5000 simulations in the PSA.

## Results

Using base case values, diagnosing and treating CBD stones using MRCP to select patients for ERCP was significantly less costly than using EUS to select patients or proceeding directly to invasive testing by ERCP ($1299 versus $1753 and $1781, respectively). Similar QALYs were accrued by each option (0.998, 0.998 and 0.997 for EUS, MRCP and direct ERCP, respectively) (Table 1). MRCP produced the same QALYs as EUS, but was less costly due to lower test costs ($356 versus $803). Using EUS or MRCP to select patients for ERCP produced negligibly more QALYs than direct ERCP because the negative impact on utility of invasive testing in patients who did not have CBD stones was avoided, but only for a short period of time. The MNB was significantly higher for MRCP compared with the other two options at a maximum willingness to pay for a QALY of $29,000 and $43,000, indicating that MRCP was preferred on cost-effectiveness grounds using base case values. As expected, the probabilistic MNBs were numerically similar to the deterministic MNBs (not shown).

**Table 1:** Base case results.

	EUS	MRCP	Direct ERCP
Costs (US$)	1,753 (1,692, 1,814)	1,299 (1,256, 1,342)	1,781 (1,724, 1,838)
QALYs	0.998 (0.998, 0.998)	0.998 (0.998, 0.998)	0.997 (0.997, 0.997)
Monetary net benefit			
$29,000	27,164 (27,103, 27,225)	27,616 (27,573, 27,660)	27,118 (27,061, 27,175)
$43,000	41,622 (41,561, 41,683)	42,074 (42,031, 42,117)	41,568 (41,511, 41,624)

EUS = Endoscopic ultrasound. MRCP = magnetic resonance cholangiopancreatography. ERCP = endoscopic retrograde cholangiopancreatography. QALY = quality adjusted life year.

Costs are in 2011/12 US$. Figures are expected values per patient with 95% confidence intervals in brackets. The point estimates are calculated using base case values of the model parameters (deterministic results). The 95% confidence intervals are derived using standard deviations calculated from the 5000 simulations in the probabilistic sensitivity analysis. The monetary net benefit is calculated at a maximum willingness to pay for a QALY of $29,000 and $43,000. Numbers may not sum due to rounding.

In the one-way sensitivity analysis the results were not sensitive to changing the base case values within the stated ranges. MRCP had the highest MNB in all situations. When the probability of developing biliary symptoms where CBD stones are present but EUS or MRCP give negative results was allowed to vary from 0 to 1 separately for both EUS and MRCP, MRCP always had the highest MNB.

**Fig 2:**
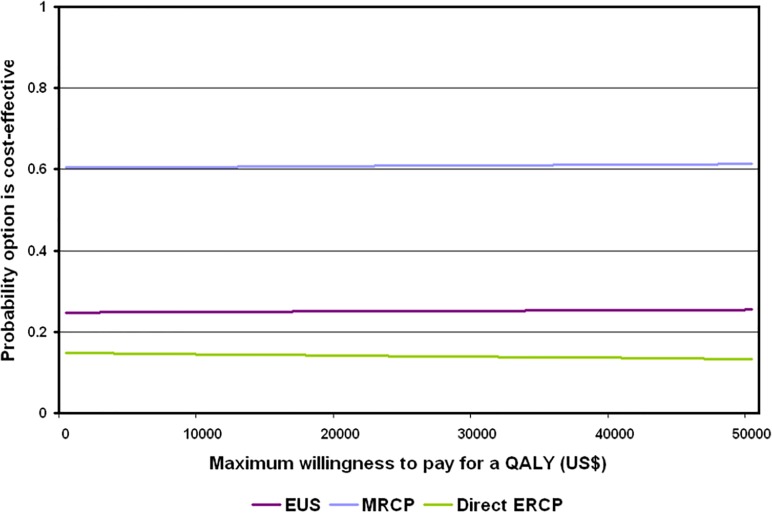
Cost-effectiveness acceptability curves at different values of the maximum willingness to pay for a QALY. Results from the cost-effectiveness acceptability analysis. MRCP had a 61.0% probability of being cost-effective at a maximum willingness to pay for a QALY of $29,000 and a 61.1% probability at a value of $43,000. For EUS the values were 25.2% and 25.3%, respectively. For direct ERCP they were 13.9% and 13.6%, respectively. EUS = Endoscopic ultrasound. MRCP = magnetic resonance cholangiopancreatography. ERCP = endoscopic retrograde cholangiopancreatography QALY = quality adjusted life year.

The cost-effectiveness acceptability curves for each treatment show that MRCP had a 61.0% probability of being cost-effective at a maximum willingness to pay for a QALY of $29,000 and a 61.1% probability at a value of $43,000 (Fig. 2). For EUS the values were 25.2% and 25.3%, respectively. For direct ERCP they were 13.9% and 13.6%, respectively.

## Discussion

### Main findings

This study compares the cost-effectiveness of using EUS or MRCP to select patients for ERCP in patients with suspected CBD stones as opposed to proceeding directly to ERCP. Initial MRCP was the most cost-effective option. It was significantly cheaper than EUS and invasive testing by ERCP. MRCP was less costly than EUS due to the lower test costs, and was less costly than direct ERCP because it avoided the costs of invasive testing in patients who did not have CBD stones. MRCP produced similar QALYs as EUS and proceeding directly to invasive testing by ERCP. EUS and MRCP avoided the utility decrement incurred by direct ERCP associated with complications of invasive testing in patients who did not have CBD stones, but in the absence of data the duration of this utility decrement was assumed to be small so similar QALYs were achieved by the three options. MRCP produced the same QALYs as EUS due to their similar sensitivity and specificity.[21] The MNBs for MRCP were significantly higher than those for EUS and ERCP at a maximum willingness to pay for a QALY of $29,000 and $43,000.

### Strengths and weaknesses

A strength of this study is that it was based on a recently published Cochrane Review that analysed in detail available evidence for the diagnostic accuracy of EUS and MRCP in patients with suspected CBD stones.[21] An extensive sensitivity analysis was performed, which showed little variation in the findings.

There are some weaknesses. First, due to lack of data, the model makes assumptions concerning the probability that EUS and MRCP do not provide diagnostic information, the probability of subsequent biliary symptoms in false negative cases where CBD stones have been missed by EUS and MRCP, and the duration of events. The model assumes an overall pretest probability of 41.8% for the presence of CBD stones. In practice, clinicians will use clinical judgement to refine their pretest estimate of CBD stones based on lab values, patient symptoms, so the subsequent sequence of testing is based on their judgement. In one way sensitivity analysis, our results were not sensitive to varying this probability within reasonable limits. The assumed values were tested in sensitivity analyses, and varying them did not alter the conclusions. Second, the model assumes the probability of successful endoscopic clearance by therapeutic ERCP following MRCP and EUS is the same, whereas the success rate may be higher following MRCP due to the extra information that it provides for the endoscopist about the anatomy of the biliary tree. There is no evidence to justify such a difference, but if this was the case then it would produce stronger findings in support of MRCP. Third, the model assumes the sensitivity and specificity of direct ERCP were both 1. ERCP is the best available test for confirmation but can misclassify the presence or absence of CBD stones.[39] While ERCP may not be 100% accurate, this is a conservative estimate to evaluate the incremental costs and benefits of using EUS or MRCP to select patients for ERCP against proceeding directly to ERCP. If a sensitivity and specificity less than 1 were assumed this would make both EUS and MRCP appear more cost-effective compared with direct ERCP, and would not affect the relative cost-effectiveness of MRCP compared with EUS. Fourth, ERCP is associated with a small risk of mortality, which has not been accounted for in the model.[40] If a small mortality risk with ERCP was assumed then EUS and MRCP would appear more cost-effective, and given the similarities between EUS and MRCP, their relative cost-effectiveness would not be affected. Fifth, this cost-effectiveness model is based on patients with suspected common bile duct stones using data from a systematic review which specifically assessed the role of EUS and MRCP in the diagnosis of common bile duct stones. Most of the included patients in the systematic review had gallstones or had previously undergone cholecystectomy for gallstones. Thus, the findings of this study are applicable only for such patients and not in people with suspected malignant obstructive jaundice. Consequently, we have not modelled the impact of missed diagnosis of malignancy in this research. Finally, the scope of this study encompasses the direct benefits and harms of MRCP and EUS in imaging CBD only. There may be other practical benefits of these modalities which would impact on their cost-effectiveness. For example, MRCP may be helpful in detecting other incidental soft tissue lesions. EUS will give information on the pancreas, perigastric and mediastinal regions which may inform management of the patient beyond CBD. Similarly it does not consider the costs of anaesthesia required in most cases for EUS. Factoring costs if anaesthesia associated with EUS into the model would further increase the cost-effectiveness of MRCP.

### Comparison with other studies

Consistent with previous studies this study finds that initial MRCP or EUS to select patients for ERCP are cost-effective compared with direct ERCP.[23–26] Unlike other studies results in this study are not sensitive to varying the probability that CBD stones were present.[23,26]

### Implications for policy and practice

In the UK MRCP should be used rather than EUS to select patients for ERCP in patients with suspected CBD stones.

The cost saving per patient found in this study could translate to $9.7 million per year for the NHS if MRCP was widely adopted to diagnose CBD stones and select patients for ERCP rather than EUS. This estimate assumes 32,300 cases of suspected CBD stones each year (based on 50 million adults in the UK,[41] a prevalence of gallstones of 15%,[21] that 2% of gallstones are symptomatic each year,[42] that 9% of these are due to CBD stones,[43–45] and a pre-test probability of CBD stones of 0.418]. [21] There is no evidence on the use of EUS versus MRCP to diagnose CBD stones in the UK[46] but if 50% of CBD stones are already diagnosed using MRCP and 50% are diagnosed using EUS then the cost saving would still be $4.8 million.[39]

### Further research

Further research is needed to evaluate the probability of inconclusive test results with EUS and MRCP, and to evaluate the management of false negative cases following EUS and MRCP.

The generalizability of these results to healthcare settings outside the UK depends on the costs of EUS and MRCP. In the UK these were $1336 and $356 respectively. Our findings were not sensitive to the costs of these tests, but further research taking account of the relative costs of these modalities outside the UK will be needed to assess generalizability.

It is likely that local availability of these modalities is a major determinant of use and limited availability could affect cost-effectiveness. MRCP is available in most hospitals, but there may be limited access outside working hours. If waiting for available MRCP results in patients needing an extra night of hospital care, this would increase the costs of this modality. In contrast, if EUS is available immediately, the endoscopist could conduct EUS initially and if positive, proceed with ERCP in the same setting, which could reduce its costs. Further research would be beneficial to explore the cost-effectiveness of improving access to MRCP.

The population expected value of perfect information (EVPI) is a measure of the maximum that the healthcare system should be willing to pay for additional research to reduce uncertainty. The EVPI is the difference between the maximum expected net benefit based on perfect information and based on current knowledge, multiplied by the size of the population that could benefit from the information.[37] Assuming 32,300 patients are suspected to have CBD stones in the UK each year, a time horizon of 10 years and a discount rate of 3.5%,[36] the EVPI was estimated to be $25.5 million at a maximum willingness to pay for a QALY of $29,000-$43,000. This is comparable with the EVPI calculated in a comparison of early versus delayed laparoscopic cholecystectomy for acute cholecystitis ($39.4-$82.9 million).[47]

### Conclusion

From the perspective of the UK NHS, MRCP was the most cost-effective test in the diagnosis of CBD stones.

## Supporting Information

S1 Table Model parameters for decision tree model and range of values used in univariate sensitivity analysis. (DOCX)

S2 Table Research Checklist CHEERS Statement. (DOC)
