# Supplementary material for: Cost-Effectiveness Analysis of Endoscopic Ultrasound versus Magnetic Resonance Cholangiopancreatography in Patients with Suspected Common Bile Duct Stones
Source: PLoS One. 2015 Mar 23;10(3):e0121699. doi: 10.1371/journal.pone.0121699 (PMC4370382; doi:10.1371/journal.pone.0121699)
Supplement: S1 Table — (DOCX) [file pone.0121699.s001.docx]

**Supplementary information. Model parameters for decision tree model and range of values used in univariate sensitivity analysis**

|  | **Base case**  **value** | **Distribution** | **Alpha** | **Beta** | **Source** | **Range** | **Treatment with**  **highest MNB** |
| --- | --- | --- | --- | --- | --- | --- | --- |
| **Probabilities** |  |  |  |  |  |  |  |
| Pr(CBDS present) [Pre-test probability] | 0.418 | Beta | 1047 | 1485 | [21] | 0.144-0.679 | MRCP |
| **Direct ERCP** |  |  |  |  |  |  |  |
| Pr(Successful endoscopic clearance by therapeutic ERCP) | 0.989 | Beta | 266 | 3 | [2] | 0.5-1 | MRCP |
| Pr(Therapeutic ERCP with complications) | 0.187 | Beta | 67 | 291 | [29] | 0.1-0.3 | MRCP |
| Pr(Open choledochotomy with complications) | 0.205 | Beta | 76 | 295 | [29] | 0.1-0.3 | MRCP |
| Pr(Diagnostic ERCP with complications) | 0.055 | Beta | 1117 | 19185 | [24] | 0-0.1 | MRCP |
| **EUS** |  |  |  |  |  |  |  |
| Pr(Complications with EUS) | 0.0003 | Uniform |  |  | [30] | 0-0.0006 | MRCP |
| Pr(With no diagnostic information) | 0.01 | Uniform |  |  | A | 0-0.02 | MRCP |
| Sensitivity | 0.950 | Beta | 107.3 | 5.7 | [21] | 0.91-0.97 | MRCP |
| Specificity | 0.970 | Beta | 119.5 | 3.7 | [21] | 0.94-0.99 | MRCP |
| Pr(Successful endoscopic clearance by therapeutic ERCP) | 0.989 | Beta | 266 | 3 | [2] | 0.5-1 | MRCP |
| Pr(Therapeutic ERCP with complications) | 0.187 | Beta | 67 | 291 | [29] | 0.1-0.3 | MRCP |
| Pr(Open choledochotomy with complications) | 0.205 | Beta | 76 | 295 | [29] | 0.1-0.3 | MRCP |
| Pr(Diagnostic ERCP with complications) | 0.055 | Beta | 1117 | 19185 | [24] | 0-0.1 | MRCP |
| Pr(Develop biliary symptoms in patients with false negative EUS) | 0.01 | Uniform |  |  | A | 0-0.02 | MRCP |
| **MRCP** |  |  |  |  |  |  |  |
| Pr(With no diagnostic information) | 0.01 | Uniform |  |  | [A] | 0-0.02 | MRCP |
| Sensitivity | 0.930 | Beta | 63.6 | 4.9 | [21] | 0.87-0.96 | MRCP |
| Specificity | 0.960 | Beta | 38.3 | 1.6 | [21] | 0.90-0.98 | MRCP |
| Pr(Successful endoscopic clearance by therapeutic ERCP) | 0.989 | Beta | 266 | 3 | [2] | 0.5-1 | MRCP |
| Pr(Therapeutic ERCP with complications) | 0.187 | Beta | 67 | 291 | [29] | 0.1-0.3 | MRCP |
| Pr(Open choledochotomy with complications) | 0.205 | Beta | 76 | 295 | [29] | 0.1-0.3 | MRCP |
| Pr(Diagnostic ERCP with complications) | 0.055 | Beta | 1117 | 19185 | [24] | 0-0.1 | MRCP |
| Pr(Develop biliary symptoms in patients with false negative MRCP) | 0.01 | Uniform |  |  | A | 0-0.02 | MRCP |
| **Utilities** |  |  |  |  |  |  |  |
| Full health | 1 |  |  |  |  |  |  |
| EUS with complications | 0.7596 | Beta | 29.24 | 9.25 | [23] | 0.6-0.9 | MRCP |
| EUS without complications | 1 |  |  |  | [23] | 0.95-1 | MRCP |
| MRCP | 1 |  |  |  | [23] | 0.95-1 | MRCP |
| Diagnostic ERCP with complications | 0.7596 | Beta | 29.24 | 9.25 | [23] | 0.6-0.9 | MRCP |
| Diagnostic ERCP without complications | 0.99 | Uniform |  |  | [23] | 0.98-1 | MRCP |
| Therapeutic ERCP with complications | 0.7596 | Beta | 29.24 | 9.25 | [23] | 0.6-0.9 | MRCP |
| Therapeutic ERCP without complications | 0.89 | Beta | 17.95 | 2.22 | [23] | 0.8-1 | MRCP |
| Open choledochotomy with complications | 0.7596 | Beta | 29.24 | 9.25 | [23] | 0.6-0.9 | MRCP |
| Open choledochotomy without complications | 0.81 | Beta | 2.31 | 0.54 | [23] | 0.6-0.9 | MRCP |
| Continuing symptoms | 0.88 | Beta | 8.60 | 1.17 | [23] | 0.8-1 | MRCP |
| **Duration of health state (weeks)** |  |  |  |  |  |  |  |
| EUS with complications | 4 | Uniform |  |  | A | 2-6 | MRCP |
| EUS without complications | 1 | Uniform |  |  | A | 0-2 | MRCP |
| MRCP | 1 | Uniform |  |  | A | 0-2 | MRCP |
| Diagnostic ERCP with complications | 4 | Uniform |  |  | A | 2-6 | MRCP |
| Diagnostic ERCP without complications | 1 | Uniform |  |  | A | 0-2 | MRCP |
| Therapeutic ERCP with complications | 4 | Uniform |  |  | A | 2-6 | MRCP |
| Therapeutic ERCP without complications | 1 | Uniform |  |  | A | 0-2 | MRCP |
| Open choledochotomy with complications | 8 | Uniform |  |  | A | 6-10 | MRCP |
| Open choledochotomy without complications | 6 | Uniform |  |  | A | 4-8 | MRCP |
| Continuing biliary symptoms | 4 | Uniform |  |  | A | 0-8 | MRCP |
| **Unit costs** |  |  |  |  |  |  |  |
| EUS with complications | 1336 | Gamma | 1 | 1336 | [31] | 500-2000 | MRCP |
| EUS without complications | 803 | Gamma | 1 | 803 | [31] | 300-1500 | MRCP |
| MRCP | 356 | Gamma | 1 | 356 | [31] | 150-1000 | MRCP |
| Diagnostic ERCP with complications | 5601 | Gamma | 1 | 5601 | [31] | 1500-7500 | MRCP |
| Diagnostic ERCP without complications | 1149 | Gamma | 1 | 1149 | [31] | 500-2000 | MRCP |
| Therapeutic ERCP with complications (per procedure) | 5601 | Gamma | 1 | 5601 | [31] | 1500-7500 | MRCP |
| Therapeutic ERCP without complications (per procedure) | 1412 | Gamma | 1 | 1412 | [31] | 500-5000 | MRCP |
| Pr(Need for second therapeutic ERCP) | 0.023 | Beta | 6 | 260 | [2] | 0-0.050 | MRCP |
| Open choledochotomy with complications | 7794 | Gamma | 1 | 7794 | [31] | 5000-10000 | MRCP |
| Open choledochotomy without complications | 6503 | Gamma | 1 | 6503 | [31] | 3000-10000 | MRCP |

Unit costs are in 2011/12 US$. CBDS = common bile duct stones. EUS = Endoscopic ultrasound. MRCP = magnetic resonance cholangiopancreatography. ERCP = endoscopic retrograde cholangiopancreatography. MNB = monetary net benefit. “A” in the Source column indicates assumption. The base case values are used to produce the deterministic results. The distributions are used to undertake the probabilistic sensitivity analysis, to produce the probabilistic results and construct the cost-effectiveness acceptability curves. In the probabilistic sensitivity analysis the parameters characterized by a uniform distribution were allowed to vary between the values shown in the Range column.
