# Supplementary material for: Cost-Effectiveness Analysis of Endoscopic Ultrasound versus Magnetic Resonance Cholangiopancreatography in Patients with Suspected Common Bile Duct Stones
Source: PLoS One. 2015 Mar 23;10(3):e0121699. doi: 10.1371/journal.pone.0121699 (PMC4370382; doi:10.1371/journal.pone.0121699)
Supplement: S2 Table — (DOC) [file pone.0121699.s002.doc]

**Research checklist CHEERS Statement**

# Cost-effectiveness analysis of endoscopic ultrasound versus magnetic resonance cholangiopancreatography in patients with suspected common bile duct stones

In accordance with recommended standards for reporting of economic evaluations,1 a completed CHEERS Statement follows.

| **Title and abstract** | | | **Page ref** |
| --- | --- | --- | --- |
| Title | 1 | We identify the study as a cost-effectiveness study in the title, and we describe the interventions being compared in the title. | 1 |
| Abstract | 2 | A structured summary is provided that conforms to the journal guidelines. | 2 |
| **Introduction** | | |  |
| Background and objectives | 3 | The broader context for the study is described in the Introduction. | 3 |
|  |  | The study question, and its relevance, is described at the end of the Introduction. | 3 |
| **Methods** | | |  |
| Target population and subgroups | 4 | The target population is described in the Methods section. | 4 |
| Setting and location | 5 | The study setting is described in the in the Methods section. | 4 |
| Study perspective | 6 | The perspective of the study is described in the Methods section. The cost components included are described in the Costs sub-section in the Methods. | 4 |
| Comparators | 7 | The options being compared are described and justified in the Methods, Model Structure sub-section. | 4 |
| Time horizon | 8 | The time horizon is stated in the Methods section. | 4 |
| Discount rate | 9 | We explain in the Methods section that due to the time horizon discounting of costs and benefits was unnecessary. | 4 |
| Choice of health outcomes | 10 | The outcome measure is described and justified in the Methods, Model Structure sub-section, with reference to the Cochrane Review and other literature that informed. | 5 |
| Measurement of effectiveness | 11b | The measure of effectiveness is described and justified in the Probabilities and Outcomes sub-sections in the Methods section. | 5-6 |
| Measurement and valuation of preference based outcomes | 12 | The methods used to obtain preferences for outcomes are described in the Outcomes sub-section in the Methods section. | 5-6 |
| Estimating resources and costs | 13b | The methods and data sources used to measure resource use and unit costs, and to calculate costs are described in the Methods section and the Costs sub-section in the Methods section. | 4-7 |
| Currency, price date, and conversion | 14 | The currency and price date is stated in the Methods section. Costs are converted to US$ for international generalizability. | 4 |
| Choice of model | 15 | The type of model used is described in the Methods section, and illustrated in Fig 1. | 4-5 |
| Assumptions | 16 | The assumptions underpinning the model are described throughout the Methods section. | 4-7 |
| Analytical methods | 17 | The analytical methods supporting the evaluation are described throughout the Methods section. | 4-7 |
| **Results** | | |  |
| Study parameters | 18 | The study parameter values, ranges, references and probability distributions are described in the Methods section, and illustrated in Table 1. | 5 |
| Incremental costs and outcomes | 19 | The costs and outcomes for each comparator are reported in the Results section. Monetary net benefits are reported. | 7 |
| Characterising uncertainty | 20b | Evaluation of uncertainty is discussed in the Sensitivity analysis sub-section in the Methods section and in the Results section. | 8 |
| Characterising heterogeneity | 21 | Not applicable. |  |
| **Discussion** | | |  |
| Study findings, limitations, generalisability, and current knowledge | 22 | Study findings, limitations, generalizability and how the findings fit with current knowledge are discussed in the Discussion section. | 8-10 |
| **Other** | | |  |
| Source of funding | 23 | The source funding source and the role of the funder are acknowledged in the submission. |  |
| Conflicts of interest | 24 | A statement in line with ICMJE recommendations is given in the submission. |  |

**Reference**

1. Husereau D, Drummond M, Petrou S, Carswell C, Moher D, Greenberg D, Augustovski F, Briggs AH, Mauskopf J, Loder E; CHEERS Task Force. Consolidated Health Economic Evaluation Reporting Standards (CHEERS) statement. *[Br Med J](http://www.ncbi.nlm.nih.gov/pubmed/23529982#%23)* 2013; **346**: f1049. doi: 10.1136/bmj.f1049.
